# Role of Perilipins in Oxidative Stress—Implications for Cardiovascular Disease

**DOI:** 10.3390/antiox13020209

**Published:** 2024-02-07

**Authors:** Mathieu Cinato, Linda Andersson, Azra Miljanovic, Marion Laudette, Oksana Kunduzova, Jan Borén, Malin C. Levin

**Affiliations:** 1 Department of Molecular and Clinical Medicine/Wallenberg Laboratory, Institute of Medicine, The Sahlgrenska Academy, University of Gothenburg, Sahlgrenska University Hospital, 41345 Gothenburg, Sweden; mathieu.cinato@wlab.gu.se (M.C.); linda.andersson@wlab.gu.se (L.A.); azra.miljanovic@wlab.gu.se (A.M.); marion.laudette@wlab.gu.se (M.L.); jan.boren@wlab.gu.se (J.B.); 2 Institute of Metabolic and Cardiovascular Diseases (I2MC), National Institute of Health and Medical Research (INSERM) 1297, Toulouse III University—Paul Sabatier, 31432 Toulouse, France; oxana.koundouzova@inserm.fr

**Keywords:** lipid droplets (LDs), perilipins (Plins), oxidative stress, reactive oxygen species (ROS), cardiovascular disease

## Abstract

Oxidative stress is the imbalance between the production of reactive oxygen species (ROS) and antioxidants in a cell. In the heart, oxidative stress may deteriorate calcium handling, cause arrhythmia, and enhance maladaptive cardiac remodeling by the induction of hypertrophic and apoptotic signaling pathways. Consequently, dysregulated ROS production and oxidative stress have been implicated in numerous cardiac diseases, including heart failure, cardiac ischemia–reperfusion injury, cardiac hypertrophy, and diabetic cardiomyopathy. Lipid droplets (LDs) are conserved intracellular organelles that enable the safe and stable storage of neutral lipids within the cytosol. LDs are coated with proteins, perilipins (Plins) being one of the most abundant. In this review, we will discuss the interplay between oxidative stress and Plins. Indeed, LDs and Plins are increasingly being recognized for playing a critical role beyond energy metabolism and lipid handling. Numerous reports suggest that an essential purpose of LD biogenesis is to alleviate cellular stress, such as oxidative stress. Given the yet unmet suitability of ROS as targets for the intervention of cardiovascular disease, the endogenous antioxidant capacity of Plins may be beneficial.

## 1. Introduction

Myocardial disease remains the leading cause of death and disability worldwide. Despite advances in medical and interventional therapy for myocardial pathologies such as ischemia, valvular heart disease, or hypertension, many surviving patients still develop heart failure. In addition, recent studies show that the incidence of heart failure is increasing in the younger population in parallel with the increasing prevalence of diabetes and obesity [1,2,3].

Oxidative stress is a common denominator in the pathogenesis of myocardial disease. Oxidative stress is the imbalance between the production of reactive oxygen species (ROS) and antioxidants in a cell. In patients with myocardial disease, oxidative stress occurs in the myocardium and is associated with left ventricular dysfunction [4,5,6]. Within the heart, oxidative stress can impair calcium handling, cause arrhythmia, and enhance maladaptive cardiac remodeling by the induction of hypertrophy and apoptosis [7].

**Figure 1:**
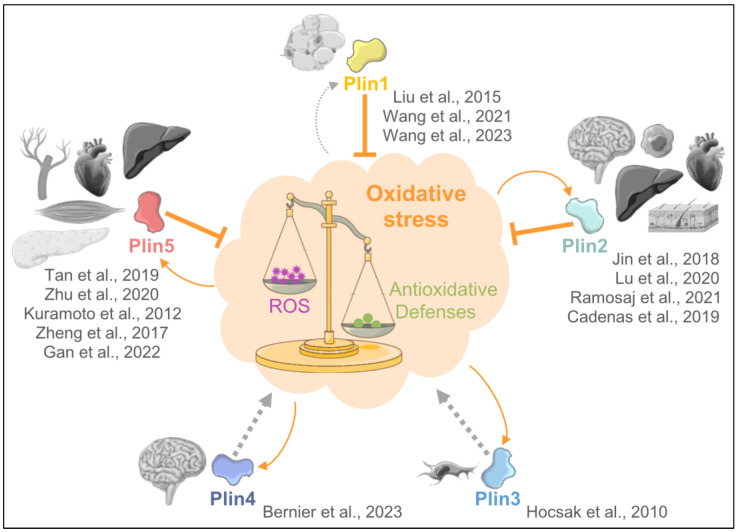
Schematic illustration of the interplay between oxidative stress and perilipins (Plins). The effect of each plin on oxidative stress is shown in thick orange lines, and the effect of oxidative stress on perilipin levels and location is shown in thin arrows. Studied organs and cells as well as references (Plin1: [8,9,10]; Plin2: [11,12,13,14]; Plin3: [15]; Plin4: [16]; Plin5: [17,18,19,20,21]) are highlighted in grey for each perilipin. Grey dotted arrows highlight the current gap in knowledge.

Currently, emerging evidence indicates that lipid droplets (LDs) play a critical role in the cellular response to oxidative stress. In this review, we focus on the role of the LD proteins perilipins (Plins) and their role in oxidative stress. We will also discuss the implications of Plins and oxidative stress in cardiovascular disease. For a schematic summary, please see Figure 1.

### 1.1. ROS within the Heart

Oxidative stress occurs when there is an excessive production of ROS in relation to antioxidant defense. ROS are oxygen-containing reactive species and comprise oxygen free radicals (e.g., superoxide anion radical O_2_^•−^, hydroxyl radicals, and peroxyl radicals) as well as non-radicals (e.g., hydrogen peroxide H_2_O_2_, hypochlorous acid, and ozone) [22].

In most cell types, and especially in cardiomyocytes, the mitochondrial electron transport chain is the main endosource of ROS production [23]. A fraction of the electrons running through the electron transport chain in the mitochondrial inner membrane are partially reduced to O_2_^•−^ and are rapidly dismutated to H_2_O_2_ by superoxide dismutase (SOD) and then further reduced to H2O by antioxidative enzymes (gluthatione peroxidase, peroxiredoxin, and catalase). In addition to mitochondria, ROS can also be generated by cytosolic sources. One of the most important sources of cytosolic ROS are the NADPH oxidases (NOX) enzymes [24]. NOX proteins produce O_2_^•−^ through NADPH electron exchange, and NOX-dependent ROS production regulates many metabolic processes and has been implicated in cardiovascular disease [25]. Furthermore, cytosolic ROS are also produced by xanthine oxidase, nitric oxide synthase, monoamine oxidase, cyclooxygenases, and cytochrome P450 enzymes [26].

The tight equilibrium between ROS production and neutralization is ensured either by the regulation of the expression/activity of enzymes producing free radicals or by the endogenous antioxidant system. The latter comprises antioxidant enzymes, such as SOD, catalase, or glutathione peroxidase, as well as small molecules, such as hydrophilic antioxidants and lipophilic radical antioxidants [27]. To protect itself from oxidative stress, the cell can activate the antioxidant response element found within the promoter region of many cytoprotective antioxidants [28]. The antioxidant response element is transcriptionally activated through nuclear translocation/accumulation and the binding of its transcription factor, Nrf2 (NF-E2-related), and thus it is responsible for the regulation of a large panel of antioxidant enzymes [29].

Under physiological conditions, ROS regulate many cellular processes when present at low concentrations, including gene expression, energetic production, substrate oxidation, hormone production, and cellular defenses [30]. Elevated ROS levels overpassing the antioxidant defense can also be beneficial in certain situations (i.e., antimicrobial defense, exercise adaptation). However, excessive ROS production leading to oxidative stress is mainly toxic, resulting in damaged cell constituents and impaired cellular function. In the heart, multiple studies have shown that ROS impair a broad range of cellular functions, including mitochondrial function and biogenesis, mitochondrial permeability transition pore opening, energy metabolism, calcium handling, excitation–contraction coupling, cardiac fibroblast activation, and cell death [31,32,33,34,35,36]. Additionally, oxidative stress due to a reduction in the antioxidant defense has also been identified as a contributing factor to cardiomyocyte dysfunction [26].

Certainly, dysregulated ROS production and oxidative stress trigger maladaptive cardiac remodeling in numerous cardiac diseases, including cardiac ischemia–reperfusion injury, arrhythmia, hypertrophy, and diabetic cardiomyopathy potentially progressing to heart failure [26,37,38].

### 1.2. Cardiac Dysfunction Promotes Metabolic Abnormalities

In cardiomyocytes, lipid homeostasis depends on a dynamic balance between fatty acid uptake from the surroundings and consumption by mitochondrial β-oxidation. Cardiac dysfunction and remodeling are known to promote metabolic abnormalities [39]. The heart has a very high energy demand and must maintain a continuous production of ATP to sustain contractile function. The healthy heart is metabolically flexible and can easily switch between different energy substrates, such as fatty acids and glucose. However, the failing heart loses this flexibility, resulting in a decreased ability to produce energy through ATP and other high-energy phosphate compounds [40,41,42], by up to 40% [43]. The energy deficit in the failing heart is associated with profound metabolic reprogramming, including an increased uptake of lipids and glucose and a subsequent accumulation of lipids [44]. It is well known that neutral lipids (triglycerides and cholesteryl esters) accumulate in LDs in the diseased heart [45]. In addition, we and others have shown that other lipid intermediates (potentially lipotoxic and/or bioactive lipids) also accumulate in the remodeling heart in response to a pathological insult [46,47,48,49,50]. In response to metabolic reprogramming, the abnormal accumulation of myocardial LDs may impact the redox state of the heart.

### 1.3. LDs and Plins in the Heart

LDs are conserved intracellular organelles found in almost all cell types [51,52]. This dynamic organelle consists of a core of neutral lipids such as cholesterol esters and triglycerides [53]. LDs store lipids that can be used as metabolic fuel and for membrane components, posttranslational protein modifications, and signaling molecules within the cell [52,54]. They can vary in size from 100 nm up to 100 µm in white adipose tissue (WAT), filling up the entire adipocyte [52,55]. The membrane that surrounds the core consists of phospholipids, cholesterol, and proteins with different functions [51,54,55,56,57].

Proteomic studies have identified more than 200 proteins that are associated with LDs [51,58]. The proteins that coat the LDs can vary between droplets within the cell, between metabolic conditions, and between cell types, and the limited capacity for proteins to bind to LDs further regulates this [52,54,59]. One of the major LD protein families are the Plins [59,60]. To date, five different Plins have been identified in mammals, Plins1–5 [58]. Plins sequester lipids by protecting LDs from lipases. Plin2 and Plin3 are ubiquitously expressed in many tissues and cells, whereas Plin1, 4, and 5 have more specialized tissue expression [54].

Within the heart, LD storage is normally low, with small and few LDs. However, abundant and enlarged LDs are found in the hearts of patients with diabetes, obesity, and metabolic syndrome, as well as with cardiovascular disease [61]. In the heart, four Plins are present (Plin2, Plin3, Plin4, and Plin5). However, Plin2 and Plin5 are by far the most abundant LD proteins in cardiomyocytes [60,62,63].

### 1.4. LDs and Oxidative Stress

Emerging evidence indicates that LDs, in addition to mere energy metabolism and lipid handling, play important roles in the cellular stress response [64,65]. Numerous reports suggest that an essential purpose of LD biogenesis is to relieve cellular lipotoxic stress, as well as oxidative stress.

Oxidative stress resulting from the overproduction of ROS often correlates with the increased biogenesis of LDs. The causal mechanisms are still not clarified, but potential mechanisms may be the activation of lipogenesis mediated by sterol regulatory element-binding protein (SREBP) as well as altered phospholipid turnover [66,67]. In addition, previous studies have shown that a reduction in a cell’s ability to form LDs may result in severe oxidative stress. Cheng et al. reported that the inhibition of DGAT1 (a major triglycerides-synthesizing enzyme; diacylglycerol-acyltransferase 1) disrupted LD accumulation and led to an increased flux of FAs to mitochondria, resulting in mitochondrial damage, oxidative stress, and apoptosis [68]. Furthermore, Bailey et al. described the antioxidant role of LDs, which limit ROS production from the peroxidation of polyunsaturated FAs in the neuroblasts of drosophila. Their results clearly indicate that LDs comprise a multifunctional organelle that controls energy metabolism, signaling molecules, and intracellular lipotoxicity [66].

The physical association between mitochondria and LDs is a growing research area. Accumulating reports show that this inter-organelle association crucially defines a metabolically distinct subset of mitochondria called peridroplet mitochondria [69]. Beyond the mere bioenergetics, peridroplet mitochondria show a distinct proteome and cristae organization as well as dynamics with reduced fusion and motility [69,70]. Although the mechanisms remain unclear, this interaction may have important implications in the antioxidative properties of LDs. In response to ROS, Tan et al. described the increased incidence of these contact sites in line with increased Plin5 expression through JNK-p38-ATF signaling. In this context, Plin5 could regulate the expression levels of mitochondrial cytochrome c oxidases and alleviate ROS production [17]. In addition, emerging evidence suggests that LDs are required for the autophagic processes [71]. Mitophagy is a selective degradation pathway for defective mitochondria in the lysosomes [72]. Yet incompletely understood, the importance of DGAT1-dependent LD biosynthesis in mitophagy may provide additional evidence on LD-dependent ROS and cell stress management [73,74].

Lastly, LDs have been found to play a key role in driving inflammation by modulating immune cell function. They can provide energy and structural components to produce inflammatory mediators such as prostaglandins, leukotrienes, and cytokines [75]. LDs also interact with the inflammasome, a multiprotein complex that activates the proinflammatory cytokines interleukin-1β (IL-1β) and interleukin-18 (IL-18) in response to a multitude of pathogen-associated molecular patterns and host-derived signals, including ROS [76]. Among all the described inflammasomes, the nucleotide-binding oligomerization domain-like receptor pyrin domain-containing 3 (NLRP3) inflammasome has been the most investigated due to its implication in a wide array of inflammatory human diseases [77]. Interestingly, NLRP3 affects mitochondrial ROS production by regulating LD formation [78]. NLRP3 activation was also found to be responsible for TREM1-mediated neuroinflammation and ROS production in microglial cells [79]. In this context, TREM1 colocalized with Plin2-positive LDs accumulated through impaired lipophagy. The LDs would thus act as a shield to this pro-oxidant factor. However, the nature of the interaction and how it affects the function of TREM1 remain to be determined. TREM1 plays a key role in oxidative stress, and its inhibition has been suggested as an anti-atherosclerotic therapy [80]. In line with a maladaptive lipid-handling profile, macrophages transitioning to an inflammatory state in human atherosclerosis display high levels of both TREM1 and Plin2 [81]. The activation of the NLRP3 inflammasome is an interesting focus for many cardiovascular injuries [82,83,84] and, in particular, in non-immune cardiac cells (i.e., cardiomyocytes and cardiac fibroblasts) [85,86]. The mechanisms underlying the role of the cardiac inflammasome in relation to lipid accumulation and oxidative stress in metabolic cardiac disease remain unknown. It may provide a better understanding of the key mediators and mechanisms underlying the switch between adaptive and maladaptive LD storage.

## 2. Plins in the Context of Oxidative Stress and Cardiac Dysfunction

### 2.1. Plin1

Plin1 is preferentially expressed in adipocytes and steroidogenic cells where it acts as a barrier to prevent LD triglycerides from hydrolysis by lipase [87]. From the current knowledge, the role of Plin1 on cardiac oxidative stress is thus limited to a perturbed metabolic crosstalk between adipose tissue and the myocardium. Indeed, an increased supply of free fatty acids to cardiomyocytes in *Plin1*-knockout mice results in the accumulation of ROS species and the induction of oxidative stress. This is suggested to be the result of an imbalanced superoxide generation and the reduced ability of antioxidants to detoxify excess ROS [8]. More recently, a dual role of Plin1 has been described in modulating the immune deficiency signaling in the fat body of the fruit fly, *Drosophila melanogaster.* Plin1 was shown to be downregulated in the early stages of the immune response, leading to the formation of large LDs and thereby taking part in an antioxidative function, efficiently eliminating ROS accumulation after bacterial infection [9]. However, in accordance with a recent report on mice, the prolonged downregulation of Plin1 during persistent immune hyperactivation in Drosophila was critical in promoting large LD-higher rate of Bmm/ATGL-mediated lipolysis leading to excessive lipotoxicity [10].

### 2.2. Plin2

Plin2 is an LD-associated protein abundantly expressed in nonadipose tissues. It is constitutively associated with intracellular LDs. Plin2 is linked to LD storage in ectopic tissues, and its increased expression is associated with numerous metabolic diseases (insulin resistance, type 2 diabetes, and cardiovascular diseases) in humans as well as in animal models [88]. Global *Plin2*-knockout mice display reduced liver triglycerides levels and are resistant to diet-induced obesity [89]. Roberts et al. performed an impressive CRISPR-Cas9 screen to identify the regulators of *Plin2* expression and stability. They identified canonical genes that control lipid metabolism as well as genes involved in ubiquitination, transcription, and mitochondrial function [90]. In addition, the expression of Plin2 has been shown to be upregulated in the context of oxidative stress. Jin et al. have shown that ROS can induce LD accumulation in hepatocytes by inducing the expression of Plin2 [11]. The endogenous upregulation of Plin2 can alleviate UVA-induced oxidative stress in dermal fibroblasts [12]. Ramosaj et al. elegantly showed that in neural progenitor/stem cells, Plin2-induced LDs generated elevated ROS production but that the higher ROS levels did not result in increased lipid peroxidation [13]. In breast cancer, upregulation of Plin2 and the promotion of lipid storage mediates the adaptation to oxidative stress [14]. An accumulation of LDs, accompanied by an increased expression of Plin2, was also observed in stress-activated microglia [91]. However, in this case, elevated Plin2 levels were supporting oxidative stress in the rostral ventrolateral medulla of stressed rats through phospholipid biosynthesis and metabolism dysregulation. In addition, Plin2 was found to play a crucial role in cerebral ischemia–reperfusion by impacting proinflammatory cytokines and the NLRP3 inflammasome [92].

Plin2 is highly expressed in the heart. In mouse heart, Plin2 is upregulated during fasting-induced steatosis [93]. *Plin2*-knockout mice had increased myocardial triglyceride levels and an increased myocardial abundance of Plin3 and Plin5 compared with littermate mice. We showed that the increased triglyceride accumulation in Plin2-deficient hearts was caused by reduced lipophagy. Thus, our results suggest that Plin2 is important for the proper hydrolysis of LDs. Western blots showed that the fusion marker mitofusin2 was significantly upregulated in Plin2-deficient hearts, but the fission marker Drp1 was not affected. The expression of mitochondrial proteins OXPHOS proteins complex IV and complex I were reduced in Plin2-deficient hearts. However, basal and maximal mitochondrial respiration was not affected by the lack of Plin2 [63]. Thus, mitochondrial function is intact in *Plin2*^−/−^ cardiomyocytes, and the increased triglyceride accumulation in *Plin2*^−/−^ cardiomyocytes is not due to differences in respiration.

Ueno et al. investigated the pathophysiological role of myocardial Plin2 by generating a transgenic mouse model with cardiomyocyte-specific overexpression of *Plin2*. *Plin2*-overexpressing hearts displayed massive triglyceride accumulation but preserved myocardial morphology and cardiac function in young mice [94]. In another overexpression model, Sato et al. found that Plin2-induced cardiac steatosis resulted in deteriorated gap junctions in the intercalated discs, impaired conduction propagation, and a higher incidence of atrial fibrillation in aged mice [95].

### 2.3. Plin3

Plin3 is ubiquitously distributed among tissues [96,97]. Plin3 binds to newly synthesized LDs but is replaced by other Plins, such as Plin2 and Plin5, when the LD matures [98]. Plin3 has the ability to move on and off the LD and is stable in the cytoplasm [96]. The depletion of hepatic Plin3 by antisense oligos suppresses hepatic steatosis and improves glucose homeostasis in mice [99]. In response to oxidative stress-induced apoptosis, Plin3 has been shown to be recruited to the mitochondria where it can protect mitochondrial membrane activity without affecting ROS generation [15].

### 2.4. Plin4

Plin4 is the least studied member of the Plin family, and knowledge about its regulation and function is still scarce. Plin4 is mainly found in preadipocytes and in membranes of newly synthesized LDs [100]. Pourteymour et al. have also shown that Plin4 is expressed in human skeletal muscle [101]. Indeed, genetic variation in the *PLIN4* gene was recently identified in an Italian cohort, resulting in the accumulation of Plin4 within muscle fibers, disrupted fiber organization, and reduced muscle contractility [102,103]. Recently, the increased expression of Plin4 during chemically induced oxidative stress has been reported [16].

### 2.5. Plin5

Plin5 is highly expressed in oxidative tissue [62] and the most studied member of the Plin family in the context of oxidative stress [104]. Cellular ROS levels have been shown to promote Plin5 expression in hepatic cells [17]. Furthermore, a recent report highlighted Plin5 as being the only Plin with a reduced expression on LDs from LPS-treated livers, suggesting that the ROS burden could also affect its location/function [105]. However, even if reports accumulate about the role of Plin5 in reducing oxidative damage, the knowledge about its regulation in cells facing an oxidative burden is still scarce. Zhu et al. showed that Plin5 regulates protection against oxidative damage by mediating the Nrf-antioxidant response element signaling pathway in pancreatic β-cells [18].

Plin5 is essential for maintaining LDs in oxidative tissues by antagonizing lipases [60,106,107]. *Plin5*-knockout mice lack detectable LDs in cardiomyocytes and have markedly reduced triglycerides accumulation in the heart [19,48]. Plin5-deficient cardiomyocytes undergo a metabolic shift by decreasing the fatty acid uptake and instead increasing the glucose uptake and are thereby able to maintain their energy metabolism. *Plin5*-knockout mice maintain a close-to-normal heart function under baseline conditions. However, during stress or myocardial ischemia, Plin5 deficiency results in myocardial reduced substrate availability, severely impaired cardiac function, and increased mortality [48]. Kuramoto et al. further showed that the production of ROS was increased in the *Plin5*^−/−^ mouse hearts, leading to a reduced heart function with age. This was, however, reduced by the administration of *N*-acetylcysteine, a precursor of an antioxidant, glutathione. In addition, Plin5-deficient mice displayed aggravated cardiac hypertrophy and elevated myocardial oxidative stress following transaortic constriction [108]. Thus, LDs prevent excess ROS production by sequestering fatty acids from oxidation and hence suppress oxidative burden to the heart [19]. Moreover, Zheng et al. have shown that Plin5 reduces oxidative stress following myocardial ischemia–reperfusion injury, through the inhibition of the lipolysis of LDs [20]. The authors showed that Plin5 deficiency increased the myocardial infarct area and reduced the heart function. Furthermore, Plin5-deficient myocardium displayed damaged mitochondria, increased ROS and malondialdehyde levels, and reduced SOD activity [20].

Plin5 provides a physical linkage to mitochondria by anchoring the mitochondria to the LD by the C-terminal region of Plin5 [109]. A deficiency in Plin5 in cardiomyocytes has also been shown to result in reduced mitochondrial function [110]. In mitochondria isolated from Plin5-deficient hearts, the oxidative capacity was reduced. However, there was no effect on the mitochondrial oxidative stress or the generation of ROS [110]. Miner et al. recently showed that FATP4 is a mitochondrial interactor of Plin5, enabling fatty acid channeling from LDs to mitochondria [111]. Kien et al. further investigated the impact of LD–mitochondria coupling by comparing a truncated form of Plin5 (that lacked the ability to couple to mitochondria) with wildtype Plin5. They found that efficient coupling between Plin5 and mitochondria had no effect on fatty acid oxidation but significantly improved the respiratory capacity of mitochondria [112]. Higher mitochondrial respiration may involve the increased production of mitochondrial ROS without deleterious effects. In this context, *Plin5* overexpression may thus enhance ROS detoxification and/or improve their usage towards beneficial pathways. Future studies of the mitochondrial interactors of Plin5 will improve the understanding of how Plin5 manages mitochondrial ROS production in pathophysiological conditions.

Additional studies have suggested a role for Plin5 in atherosclerosis and oxidative stress. Plin5 deficiency leads to accelerated atherosclerosis progression and oxidative stress in *ApoE^−/−^* mice [113]. The inactivation of Plin5 in macrophages resulted in elevated inflammation and oxidative stress [113]. Moreover, Plin5 also regulates vascular smooth muscle cell proliferation by modulating ROS generation [21].

In humans, genetic variation in *PLIN5* is associated with impaired cardiac function after myocardial ischemia. Patients carrying the allele rs884164 are at higher risk of cardiovascular morbidity and mortality after myocardial ischemia [48].

### 2.6. Transcriptional and Posttranslational Regulation of Plins in Response to ROS

While it is well recognized that oxidative stress leads to the increased expression of Plins in various tissues, the mechanisms of the transcriptional and posttranslational regulation of Plins by oxidative stress need further investigation. The PPAR family of transcription factors is key for the regulation of most of mammalian Plins (i.e., Plin1–2 and 4–5) [114]. In combination with specific transcriptional cofactors, such as PGC1 family members, the three PPAR isoforms (α, β/δ, and γ) regulate the differential cell-specific activations of Plin1, 2, 4, and 5 in different tissues. ROS levels can regulate transcriptional activity, including PPARγ function [115,116]. With transcriptional regulation, the stability of Plins is the determining factor for their tissue-specific expression levels and function. One of the most important targets of redox-based modifications is the redox-sensitive thiols of cysteines [117]. The C-terminus of Plin1 protein is a relatively conserved hydrophobic domain with five cysteine residues that have been shown to be sulfhydrated, thereby stabilizing the protein [118]. The recent development of a broad and quantitative analysis of the cysteine proteome in living tissues [119] represents an exciting approach to better understand how persistent ROS exposure could affect Plin stability and regulate their expression during oxidative stress. In addition, several reports show that posttranslational modifications of Plins are crucial for their location and function [59,120,121]. Unbiased screening approaches could facilitate more thorough investigations of the dynamic of oxidative stress-linked posttranslational modifications of Plins. Studies of human single-nucleotide polymorphisms in combination with genetically engineered mice would therefore help the understanding of the specific role of each Plin in the management of cardiac oxidative stress.

## 3. Concluding Remarks and Perspectives

One of the limitations in studying the isoform-specific functions of Plins in ROS management is the tight complexity in the regulation of Plins. It has been established that most mouse-engineered models for one specific Plin result in consequent regulation and compensation by other members of the family. For example, Plin3 and Plin5 are increased in *Plin2*-knockout mice, suggesting that they may be able to compensate for some Plin2 functions that are lost [63,122]. Above all, all Plins regulate LD turnover and access to lipases [59], resulting in the dysregulation of metabolic by-products of Plin-regulated lipolysis in genetically engineered models. Consequently, the feedback regulation of non-targeted Plins may be promoted, complicating the interpretation of their isoform-specific role.

Even though extensive experimental studies suggest that ROS are suitable targets for the intervention of cardiovascular disease, clinical trials have unfortunately shown negative results [123]. Contributing factors to the lack of clinical effects may be the different primary pathophysiologic mechanisms depending on the etiology of the disease and the large number of exogenous and endogenous antioxidant players, which implies a high interpatient variability [124]. ROS generation and interactions with other signaling molecules have been shown to occur in a compartmentalized manner, and thus there are still opportunities for novel approaches, especially targeting the endogenous antioxidant capacity. Preclinical models have provided strong evidence that LDs can function as ROS scavengers; however, knowledge about the specific role of LD proteins such as Plins in this context needs to be further investigated.

Specific modulation of Plin levels is challenging and limits the therapeutic options for ROS mitigation. However, there are increasing reports showing that increased levels of Plin2 or Plin5 reduce cellular oxidative damage. Therefore, testing the endogenous antioxidant capacity of Plins to alleviate oxidative stress in heart failure appears to be an exciting opportunity in ROS reduction during cardiovascular disease. The difficulties in specifically modulating Plin levels represent the major limitation to success in this unmet scientific challenge. However, a recent report showed that LDs can be released in the extracellular space and exchanged between cells [125]. This raises the question of the biological role of this mechanism and the potential role of LD proteins in extracellular communication. This has huge potential for the use of LDs as extracellular vesicles as novel drug delivery vehicles but most importantly as a therapy itself. In this context, promising work from Zhao et al. validated that Plin-coated artificial LDs could be taken up by cells, significantly reducing hydrogen peroxide-induced ROS and alleviating cellular lipotoxicity caused by excess fatty acids [126].

Recently, we have shown that cardiac-specific *Plin5* overexpression promotes physiological-like hypertrophy with preserved/improved cardiac function [127]. This study emphasized the therapeutic potential of the Plins to maintain cardiac physiology in challenging settings. Contrary to maladaptive remodeling, physiological cardiac hypertrophy is considered harmless, completely reversible, and mostly occurs in response to increased workload such as exercise. Mounting evidence supports the notion that ROS as well as Plin levels are tightly regulated during exercise-induced adaptations [128,129,130,131,132]. Further investigations are needed to elucidate the interplay between Plins and ROS in the context of exercise and physiological hypertrophy.

## Data Availability

No data were used for the research described in this article.
